# AbaA Regulates Conidiogenesis in the Ascomycete Fungus *Fusarium graminearum*

**DOI:** 10.1371/journal.pone.0072915

**Published:** 2013-09-10

**Authors:** Hokyoung Son, Myung-Gu Kim, Kyunghun Min, Young-Su Seo, Jae Yun Lim, Gyung Ja Choi, Jin-Cheol Kim, Suhn-Kee Chae, Yin-Won Lee

**Affiliations:** 1 Department of Agricultural Biotechnology and Center for Fungal Pathogenesis, Seoul National University, Seoul, Republic of Korea; 2 Department of Microbiology, Pusan National University, Busan, Republic of Korea; 3 Eco-friendly New Materials Research Group, Research Center for Biobased Chemistry, Division of Convergence Chemistry, Korea Research Institute of Chemical Technology, Daejeon, Republic of Korea; 4 Department of Biochemistry, Paichai University, Daejeon, Republic of Korea; Soonchunhyang University, Republic of Korea

## Abstract

*Fusarium graminearum* (teleomorph *Gibberella zeae*) is a prominent pathogen that infects major cereal crops such as wheat, barley, and maize. Both sexual (ascospores) and asexual (conidia) spores are produced in *F. graminearum*. Since conidia are responsible for secondary infection in disease development, our objective of the present study was to reveal the molecular mechanisms underlying conidiogenesis in *F. graminearum* based on the framework previously described in *Aspergillus nidulans*. In this study, we firstly identified and functionally characterized the ortholog of AbaA, which is involved in differentiation from vegetative hyphae to conidia and known to be absent in *F. graminearum*. Deletion of *abaA* did not affect vegetative growth, sexual development, or virulence, but conidium production was completely abolished and thin hyphae grew from abnormally shaped phialides in *abaA* deletion mutants. Overexpression of *abaA* resulted in pleiotropic defects such as impaired sexual and asexual development, retarded conidium germination, and reduced trichothecene production. AbaA localized to the nuclei of phialides and terminal cells of mature conidia. Successful interspecies complementation using *A. nidulans* AbaA and the conserved AbaA-WetA pathway demonstrated that the molecular mechanisms responsible for AbaA activity are conserved in *F. graminearum* as they are in *A. nidulans*. Results from RNA-sequencing analysis suggest that AbaA plays a pivotal role in conidiation by regulating cell cycle pathways and other conidiation-related genes. Thus, the conserved roles of the AbaA ortholog in both *A. nidulans* and *F. graminearum* give new insight into the genetics of conidiation in filamentous fungi.

## Introduction

*Fusarium graminearum* (teleomorph *Gibberella zeae*), a homothallic ascomycete fungus, is the major causal agent of head blight disease in wheat, barley, and rice as well as ear and stalk rot in maize worldwide [1]. Severe epidemics of these diseases result in not only yield losses but also mycotoxicoses in both humans and livestock through the production of mycotoxins in infected crops [2], [3]. Both sexual (ascospores) and asexual (conidia) spores are produced in *F. graminearum*, and ascospores are the primary inocula during the period of flowering [1], [4]. Ascospores of *F. graminearum* are produced and discharged from the perithecia, i.e., fruiting bodies, and the initial structures or associated hyphae of the perithecia are the survival structures for overwintering [5], [6]. Conidia are produced from the sporodochia on infected crops and are responsible for secondary infection [6]. Moreover, modified conidia (chlamydospores or chlamydospore-like structures) are proposed to be other survival structures [1], [7], [8].

Considering the importance of conidia and ascospores in the life cycle of *F. graminearum*, these biological processes should be precisely regulated temporally and spatially. To date, many genes related to various biological and biochemical functions have been known to be important for sexual development and virulence in *F. graminearum* [9], [10], [11], [12], [13], [14], [15], [16], [17], [18], [19], [20], [21], [22], [23], [24], [25], [26], [27]. However, there is scant information on conidiogenesis and little is known regarding the molecular mechanism of conidium production in this fungus. Most of the previously reported mutant phenotypes that are related to conidium production or morphogenesis seem to be derived from indirect effects of broken cellular homeostasis because most of the genes responsible for these mutant phenotypes have pivotal roles in major cellular processes [9], [15], [17], [18], [25], [28]. Recently, FgStuAp and Htf1 transcriptional regulators have been shown to be required for the formation of coniophores and phialides in *F. graminearum* [16], [29].

Many researchers have long tried to understand conidiogenesis in the model filamentous fungi *Neurospora crassa* and *Aspergillus nidulans* [30], [31], [32], [33], [34]. In *A. nidulans*, decades of research have provided an in-depth understanding of the genetic, molecular, and biochemical processes in asexual development; moreover, upstream development activators (UDAs) and the central development pathway (CDP) have been known to regulate conidiogenesis [35], [36]. Through *fluffy* mutant analysis, UDA genes have been characterized and their regulatory networks have been well formulated [36]. Downstream CDP proteins have stage-specific functions during conidiogenesis. BrlA and AbaA comprise the pathway required for differentiation from vegetative hyphae to conidia [37], [38], [39]. WetA also plays an important role in cell wall synthesis, which is related to conidium maturation [40]. In addition, the sensory and regulatory roles of the velvet complex that affect differentiation have been well studied [35], [41]. In phytopathogenic fungi, large-scale forward genetic approaches to find genes related to conidiation have been attempted, especially in *Magnaporthe oryzae* [42], [43]. Furthermore, a recent genome-wide functional analysis of transcription factors and kinases of *F. graminearum* has broadly expanded our understanding of fungal biology including conidium production [44], [45].

The aims of this study were to determine whether the conidiogenesis-related pathway of *A. nidulans* is conserved in *F. graminearum* and to identify the potential target genes for crop disease control. In this study, we successfully identified and functionally characterized the AbaA ortholog, which was previously considered as absent in *F. graminearum*. We also studied the regulatory mechanisms of AbaA through transcriptomic analysis.

## Methods

### Fungal strains and media

The *F. graminearum* Z-3639 strain was used as the wild-type strain in this study [46], and the other transgenic mutants derived from this strain are listed in Table 1. For genomic DNA (gDNA) isolation, each strain was inoculated in 5 ml of complete medium (CM) at 25°C for 3 days on a rotary shaker (150 rpm). For fungal sporulation, conidia of all strains were induced on yeast malt agar (YMA) [47] and in carboxymethyl cellulose (CMC) medium [48]. A minimal medium containing 5 mM agmatine (MMA) was used to evaluate trichothecene production [49]. The other media used in this study were made and used according to the *Fusarium* Laboratory Manual [1]. These wild-type and transgenic strains were stored in a 20% glycerol stock at −80°C.

**Table 1:** *F. graminearum* strains used in this study.

Strain	Genotype	Source or reference
Z-3639	*Fusarium graminearum* wild-type	[75]
A4	*Aspergillus nidulans* wild-type	FGSC^a^
hH1-GFP	*hH1::hH1-gfp-hyg*	[53]
mat1g	Δ*mat1-1-1::gen*; *hH1::hH1-gfp-hyg*	[53]
mat1r	Δ*mat1-1-1::gen*; *hH1::hH1-rfp-hyg*	[18]
Δ*abaA*	Δ*abaA::gen*	This study
Δ*abaA*-g	Δ*abaA::gen; hH1::hH1-gfp-hyg*	This study
AbaAc	*ΔabaA::abaA-gfp-hyg*	This study
AnAbaAc	*ΔabaA::AnabaA-hyg*	This study
AbaA-OE	*abaA::gen- P_ef1α_ -abaA*	This study
AbaA-OE-g	*abaA::gen- P_ef1α_ -abaA; hH1::hH1-gfp-hyg*	This study
AbaAcr	*ΔabaA::abaA-gfp-hyg; hH1::hH1-rfp-gen*	This study

a FGSC, Fungal Genetics Stock Center.

### Nucleic acid manipulations, PCR primers, and DNA sequencing

The gDNA was extracted as previously described [1]. Restriction endonuclease digestion, agarose gel electrophoresis, gel blotting, and DNA blot hybridization were performed in accordance with standard techniques [50]. The polymerase chain reaction (PCR) primers (Table S1) used for this study were synthesized by an oligonucleotide synthesis facility (Bionics, Seoul, Korea). DNA sequencing was performed with an ABI 3730xl DNA analyzer by Macrogen Inc. (Seoul, Korea), and the sequences were set against the *F. graminearum* Genome Database [51] and the *Fusarium* Comparative Database at the Broad Institute (http://www.broadinstitute.org/annotation/genome/fusarium_graminearum).

### Rapid amplification of cDNA ends (RACE)-PCR

We determined the *abaA* open reading frame (ORF) using RACE-PCR. A previously constructed cDNA library was used for the RACE-PCR [18]. Four fragments located around the *abaA* ORF were amplified with pPRN3-N-For/AbaA-RACE-7, AbaA-RACE-1/AbaA-RACE-4, AbaA-RACE-2/AbaA-RACE-3, and AbaA-RACE-5/pPRN3-N-Rev primers and then directly sequenced.

### Genetic manipulations and fungal transformations

A DNA construct for targeted gene deletion and complementation was amplified by the double-joint (DJ) PCR method, as previously described [52]. Briefly, the 5′- and 3′-flanking regions of the *abaA* gene and a geneticin resistance cassette (*gen*) were amplified from Z-3639 and pII99, and fused by DJ PCR under the PCR conditions previously described [53]. To complement the *abaA* deletion mutant, the DNA fragment carrying the native promoter and the *abaA* ORF was fused with green fluorescent protein gene (*gfp*), and the hygromycin resistance cassette (*hyg*) was amplified with pIGPAPA-sGFP F/HYG-F1 primers from the pIGPAPA vector [54]. Fungal transformation was performed as previously described [12].

For interspecies complementation using *A. nidulans abaA* (*AnabaA*), the *abaA* ORF, which was amplified with AnAbaA-For/AnabaA-Rev hyg primers from the gDNA of *A. nidulans*, was fused with DNA fragments amplified from gDNA of the *F. graminearum* wild-type strain with AbaA-5F/AbaA-5R AnabaA and AbaA-3F/AbaA-3R primers, respectively. The *hyg* gene was amplified with pBCATPH-comp-3 R/Gen-for primers from the pBCATPH vector [55]. The subsequent procedures for the third round of PCR and transformation were the same as for complementation using the *abaA* gene of *F. graminearum*.

To generate mutants overexpressing *abaA*, the 5′-end of *abaA* was amplified by primer pairs AbaA-5F/AbaA-5R OE and AbaA-3F OE/AbaA-3R OE, respectively. The *gen-P_ef1α_* construct, containing the elongation factor 1α promoter (*P_ef1α_*) from *F. verticillioides*, was amplified from pSKGEN [56] with Neo-for new/EF Pro-Rev new primers. Three amplicons were fused by DJ PCR as described above, and a final PCR product was amplified by the nested primers AbaA-5N and AbaA-3N OE.

### Conidium production and morphology

After each strain was incubated in 50 ml of CM for 72 h at 25°C on a rotary shaker (150 rpm), mycelia of each strain were harvested and washed twice with distilled water. To induce conidiation, harvested mycelia were spread on YMA and incubated for 48 h at 25°C under near UV light (wavelength: 365 nm, HKiv Import & Export Co., Ltd., Xiamen, China), and then conidia were collected with distilled water, filtered through cheese cloth, washed, and resuspended in distilled water to 1×10^5^ conidia/ml. After inoculating a 10-µl conidium suspension (1×10^5^ conidia/ml) of each strain in 5 ml of CMC and incubation for 72 h at 25°C on a rotary shaker (150 rpm), the number of conidia produced was counted to measure conidium production with a hemocytometer (Superior, Marienfeld, Germany). For observation of conidium morphology, the conidia produced by each strain on YMA or CMC were harvested and differential interference contrast (DIC) images were obtained with a DE/Axio Imager A1 microscope (Carl Zeiss, Oberkochen, Germany).

### Quantitative real time (qRT)-PCR

Total RNA of the wild-type and Δ*abaA* strains were extracted from the vegetative stage at 72 h after inoculation in CM liquid, and then the cultures at 6 h and 12 h after asexual induction were extracted using an Easy Spin Total RNA Extraction Kit (iNtRON Biotech, Seongnam, Korea). First-strand cDNA was synthesized with SuperScriptIII reverse transcriptase (Invitrogen, Carlsbad, CA, USA). qRT-PCR was performed with a SYBR Green Supermix (Bio-Rad, Hercules, CA, USA) and a 7500 real-time PCR system (Applied Biosystems, Foster City, CA, USA). For normalization, the endogenous housekeeping gene cyclophilin (*cyp1*; Broad Institute ID: FGSG_07439.3) was used [22]. The PCR assays were repeated three times with two biological replications. The threshold cycle (Δ*C_T_*) value of gene expression was subtracted from the Δ*C_T_* value of each sample to obtain a ΔΔ*C_T_* value. The transcript level relative to the calibrator was expressed as 2^−ΔΔ*CT*^ [57].

### Fertility test, virulence test, and trichothecene analysis

Mycelia grown on carrot agar for 5 days were mock-fertilized with 2.5% Tween 60 solution to induce sexual reproduction as previously described [1]. For outcrosses, mycelia of the female strain grown on carrot agar plates were fertilized with 1 ml of a male strain conidium suspension (1×10^6^ conidia/ml). After sexual induction, the fertilized cultures were incubated for 7 days under near UV light (HKiv Import and Export Co., Ltd.) at 25°C.

The virulence test was performed as previously described [18]. In brief, 10 µl of conidium suspension (1×10^5^ conidia/ml) was injected into a center spikelet of wheat cultivar “Eunpamil” head at midanthesis. Since *abaA* deletion mutants did not produce conidia, the mycelia suspension was used for inoculation. After inoculation, inoculated plants were placed in a humidity chamber for 3 days and transferred to a greenhouse; then, head blight symptoms were checked after 18 days.

Deoxynivalenol (DON) and 15-acetyldeoxynivalenol (15-AcDON) production from MMA was measured with a Shimadzu QP-5050 gas chromatograph-mass spectrometer (GC-MS, Shimadzu, Kyoto, Japan) with a selected ion monitoring and quantified on the basis of the biomass produced by each strain [18]. Five agar blocks (diameter, 5 mm) from a 5-day-old CM agar of each strain were used for inoculation because *abaA* deletion mutants were unable to produce conidia. The experiment was repeated three times.

### Microscopic observation

To observe localization of AbaA and histone H1 (hH1) in the nuclei, the mat1r strain [18] was fertilized with the AbaAc strain. Ascospores carrying both *abaA-gfp-hyg* and *hH1-red fluorescent protein (rfp)-gen* were selected using antibiotic resistance and confirmed by PCR. Localization was observed in cultures from CM, minimal medium (MM), and CMC. Chitin staining was conducted by adding Calcofluor white stock solution (10 mg/ml; Sigma, 18909) to mycelia samples on slide glasses as previously described [58]. Microscopic observation was performed with a DE/Axio Imager A1 microscope (Carl Zeiss) using filter set 38HE (excitation 470/40; emission 525/50) for GFP, filter set 15 (excitation 546/12; emission 590) for RFP, and filter set 49 (excitation 356; emission 445/50) for Calcofluor white.

### RNA sequencing and bioinformatic analysis

RNA-sequencing libraries were created using the Illumina TruSeq™ RNA sample prep kit with no modifications to the standard low-throughput protocol. Sequencing was performed on an Illumina HiSeq2000 instrument using the reagents provided in the Illumina TruSeq PE Cluster kit V3-cBot-HS and the TruSeq SBS Kit-HS (200 cycles) kit. The data discussed in this publication have been deposited in NCBI's Gene Expression Omnibus [51] and are accessible through GEO Series accession number GSE 46133 (http://www.ncbi.nlm.nih.gov/geo/query/acc.cgi?acc=GSE46133). The relative transcript abundance was measured in reads per kilobase of exon per million mapped sequence reads (RPKM) [59]. The log_2_ ratios of the RPKM values were used to identify differentially expressed genes.

### Promoter and pathway analysis

All information on the *F. graminearum* sequences and annotation were obtained from the *F. graminearum* Genome Database (http://mips.helmholtz-muenchen.de/genre/proj/FGDB/) [51]. Resource version 32 (ftp://ftpmips.gsf.de/fungi/FGDB/v32/) was used for gene annotation. From this genome sequence and gene annotation, 500-bp upstream sequences were collected from all predicted genes and surveyed for finding CATTCY motifs in these regions [60]. For the pathway analysis, the KEGG PATHWAY database was downloaded from the Kyoto Encyclopedia of Genes and Genomes (http://www.genome.jp/kegg/) and then it was combined with RNA-sequencing data.

## Results

### Identification of *abaA* in *F. graminearum*

**Figure 1:**
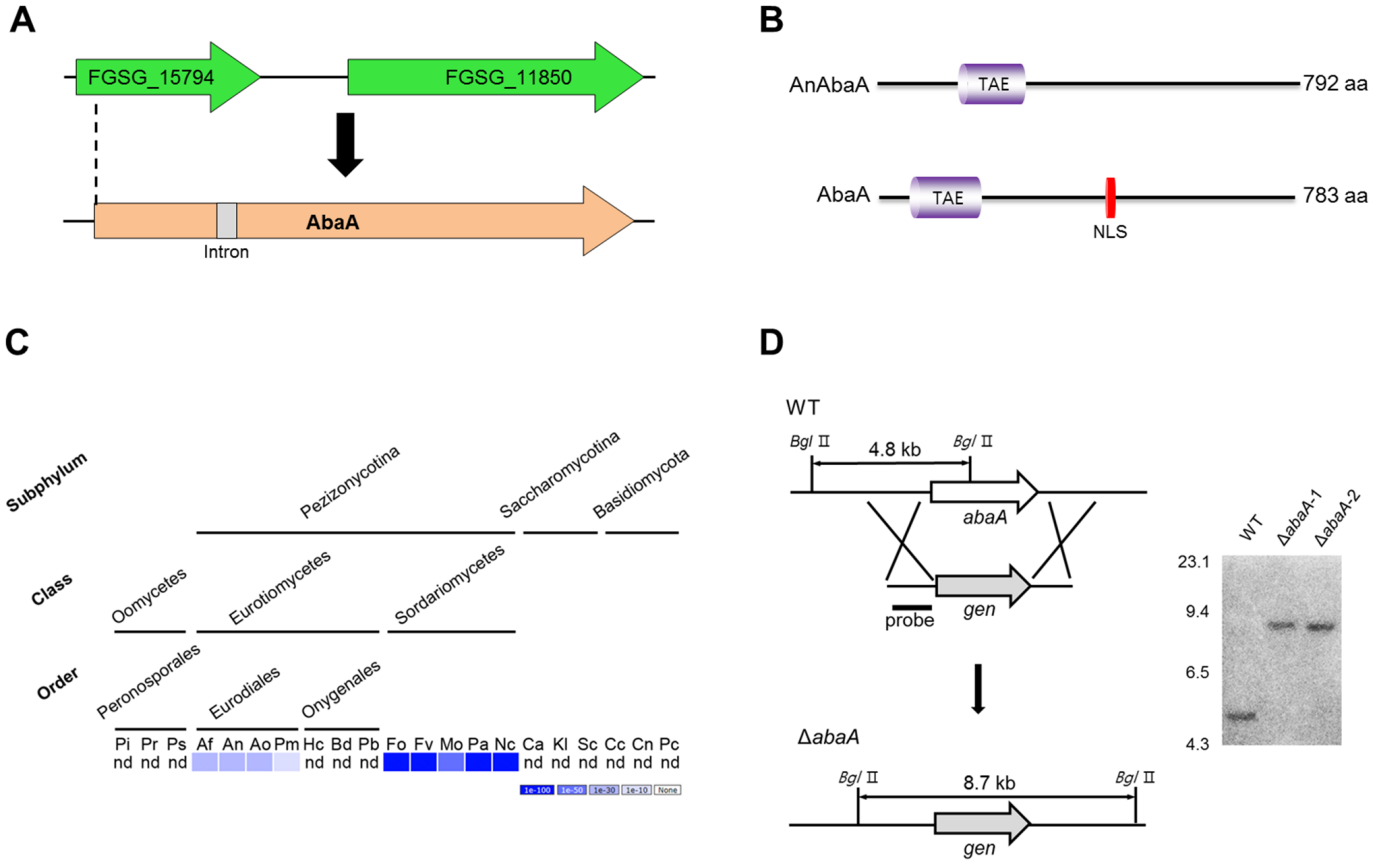
Identification and deletion of *abaA* in *F.* *graminearum*. (A) Re-annotation of the *abaA* ortholog gene in *F. graminearum*. cDNA sequencing analysis revealed an exact *abaA* open reading frame with an intron. (B) Comparison of AbaA orthologs between *A. nidulans* and *F. graminearum*. A schematic representation of the protein orthologs shows the location and alignment of conserved domains. The TAE and TEA/ATTS domains (IPR000818) predicted with InterProScan; NLS, nuclear localization signal predicted by using NLStradamus [62]. (C) Distribution of AbaA homologs in representative fungal species. The distribution image was constructed using the BLASTMatrix tool that is available on the Comparative Fungal Genomics Platform (http://cfgp.riceblast.snu.ac.kr/) [74]. Pi, *Phytophthora infestans*; Pr, *P. ramorum*; Ps, *P. sojae*; Af, *Aspergillus fumigatus*; An, *A. nidulans*; Ao, *A. oryzae*; Pm, *Penicillium marneffei*, Hc, *Histoplasma capsulatum*; Bd, *Blastomyces dermatitidis*; Pb, *Paracoccidioides brasiliensis*; Fo, *Fusarium oxysporum*; Fv, *F. verticillioides*; Mo, *Magnaporthe oryzae*; Pa, *Podospora anserine*; Nc, *Neurospora crassa*; Ca, *Candida albicans*; Kl, *Kluyveromyces lactis*; Sc, *Saccharomyces cerevisiae*; Cc, *Coprinus cinereus*; Cn, *Cryptococcus neoformans*; Pc, *Phanerochaete chrysosporium*; nd, not detected. (D) Targeted deletion of the *abaA* gene. The *abaA* gene was deleted from the *F. graminearum* wild-type genome. Left panel, schematic representation of the homologous gene recombination strategy used to generate the *abaA* deletion mutants. Right panel, Southern blot analysis. Sizes of the DNA standards used are indicated in kilobases to the left of the blot.

Previous analysis of fungal genomes for orthologs of developmental regulators known in *A. nidulans* to participate in conidiogenesis showed that AbaA and BrlA lack of orthologs in the *F. graminearum* genome [61]. The BLASTp search of AbaA of *A. nidulans* (*An*AbaA) against the *F. graminearum* genome using the *Fusarium* Comparative Database (http://www.broadinstitute.org/annotation/genome/fusarium_graminearum) provided the FGSG_11851.3 locus encoding 111 amino acids (6% identity). Recently, the *F. graminearum* Genome Database [51] re-annotated FGSG_11851.3 as FGSG_15794, which encodes 173 amino acids and contains a TEA/ATTS (IPR000818) DNA-binding domain. *An*AbaA also contains the TEA/ATTS (IPR000818) domain. Further sequencing analysis of the flanking regions of the FGSG_15794 locus and the RACE-PCR results indicated that some parts of FGSG_157941 and FGSG_11850 constitute a single gene (*abaA*) with an intron and that *abaA* encodes 783 amino acids with 20% identity to *An*AbaA (Figure 1A). The AbaA of *F. graminearum* contains a nuclear localization signal (NLS), whereas *An*AbaA does not have a detectable NLS [62] (Figure 1B). The AbaA sequence was deposited in the GenBank database [63] as accession number KC825340. BLASTMatrix analysis result showed that AbaA is only conserved in the Eurodiales order and the Sordariomycetes class (Figure 1C).

### Phenotypes of *abaA* deletion mutants

To investigate the function of *abaA*, we performed targeted gene deletion in *F. graminearum*. The ORF of *abaA* was successfully replaced with *gen* by homologous recombination and confirmed by Southern blot analysis (Figure 1D).

**Figure 2:**
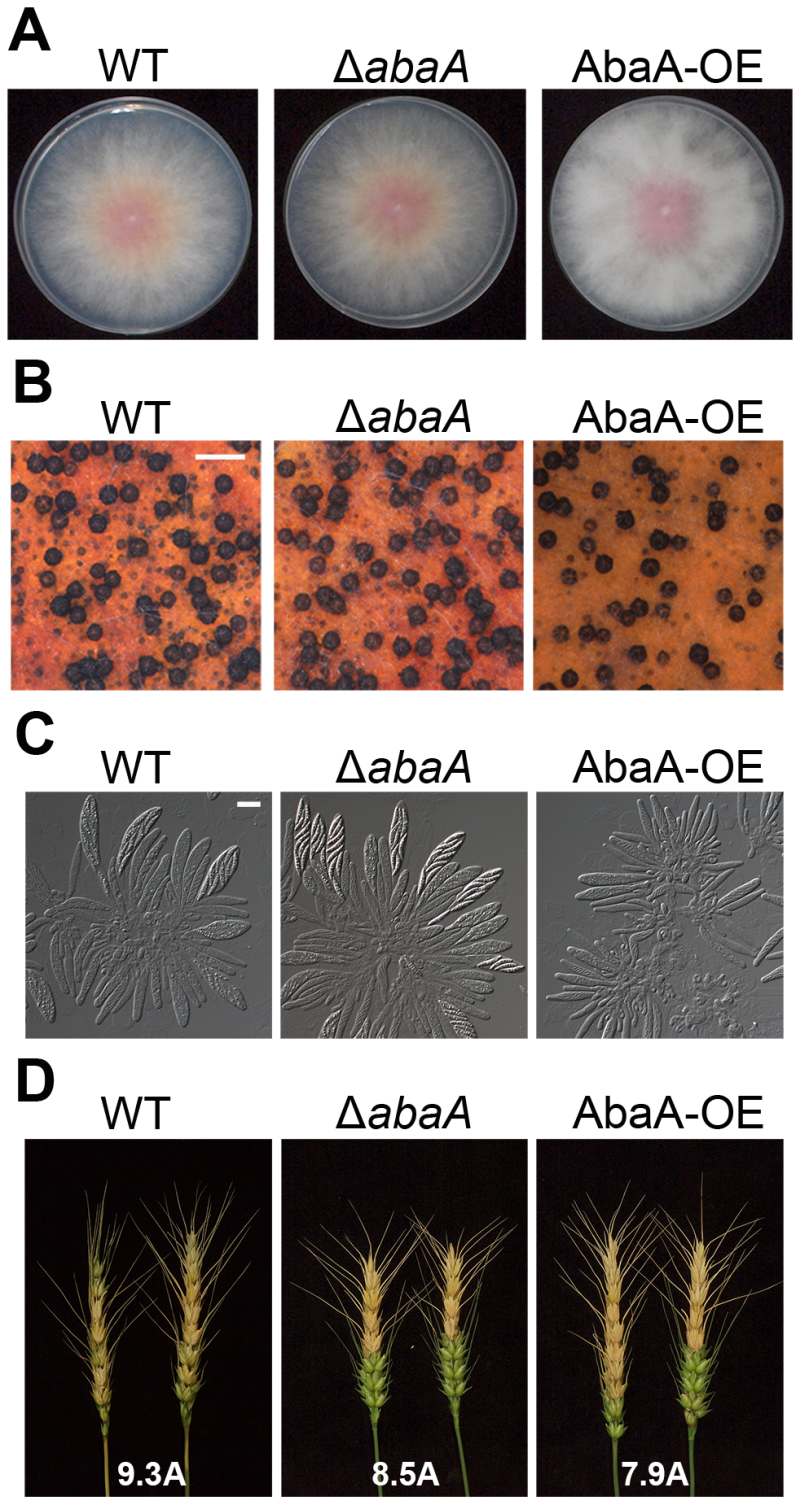
Phenotypic analyses of *abaA* deletion mutants. (A) Mycelial growth of *F. graminearum* strains on complete media (CM). Pictures were taken 5 days after inoculation on CM. (B) Perithecium formation of *F. graminearum* strains on carrot agar. Pictures were taken 7 days after sexual induction from carrot agar. Scale bar = 500 µm. (C) Asci rosettes of *F. graminearum* strains. Imaging was performed 7 days after sexual induction. (D) Virulence on wheat heads. A center spikelet of each wheat head was injected with 10 µl of a conidium suspension. Pictures were taken 21 days after inoculation. Values with different letters are significantly different (*p*<0.05) based on Tukey's test. Scale bar = 20 µm. WT, *F. graminearum* wild-type strain Z-3639; Δ*abaA*, *abaA* deletion mutant; AbaA-OE, transgenic strain with the abaA promoter replaced with the *ef1α* promoter.

**Figure 3:**
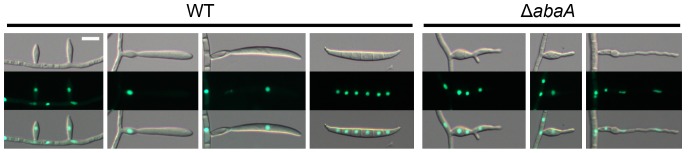
Morphology of conidiophores of *F.* *graminearum* strains. Morphology of conidiophores of *F. graminearum* strains in carboxymethyl cellulose (CMC) medium. Pictures were taken 1 to 3 days after conidium induction. Scale bar = 10 µm. WT, *F. graminearum* wild-type strain Z-3639; Δ*abaA*, *abaA* deletion mutant; AbaA-OE, transgenic strain with the abaA promoter replaced with the *ef1α* promoter.

Radial growth, sexual development, and virulence of *abaA* deletion mutants were not significantly different compared to the wild-type strain (Table 2 and Figure 2). Inoculation of the Δ*abaA* hyphae suspension caused similar disease symptoms as that of the wild-type strain (Figure 2D). To visualize nuclei during conidiophore formation *in vivo*, Δ*abaA*-g strains (Δ*abaA::gen*; *hH1::hH1-gfp-hyg*) were generated by an outcross between mat1g [53] and Δ*abaA* strains. In *F. graminearum* conidiogenesis, the wild-type strain (hH1-GFP) initially produced phialides from the hyphae, and mature phialides continuously produced conidia with 2–6 cells (Figure 3). Deletion of the *abaA* gene completely abolished conidium production in conidium induction media (CMC and YMA). In addition, *abaA* deletion mutants produced extremely immature phialides with abnormal morphology. Although some phialides seemed to generate conidia at the beginning, they germinated and resulted in thin hyphae (Figure 3).

**Table 2:** Mycelial growth and trichothecene production of *F. graminearum* strains.

Strain	Radial growth (mm)^a^	Biomass (mg/ml)^b^	Total trichothecenes (µg/g)^c^
WT	85.7	160.0	1,723
Δ*abaA*	85.5	144.7	1,704
AbaAc	84.2	165.3	2,112
AnAbaAc	84.3	178.7	2,328
AbaA-OE	86.5	265.3**	694**

a Radial growth was measured after a 4-day incubation on complete media (CM).

b Biomass was measured after a 3-day incubation in complete media (CM).

c DON and 15-AcDON concentrations were measured after a 7-day incubation in a minimal medium containing 5 mM agmatine.

** Asterisk indicates data differed significantly (*p*<0.01) based on Tukey's test.

### Complementation analysis

**Figure 4:**
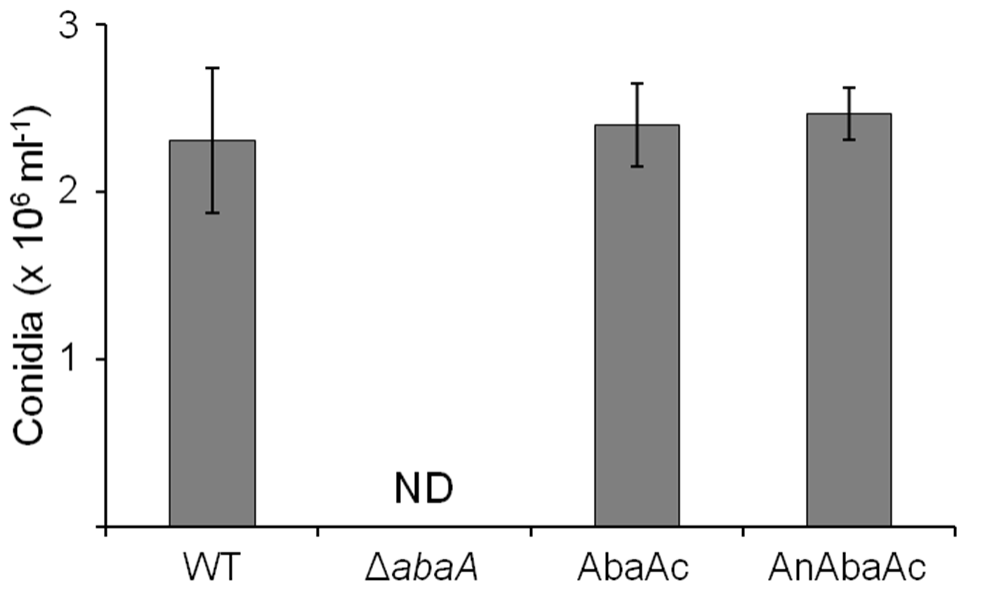
Conidiation of *F.* *graminearum* strains. Conidiation was measured by counting the number of conidia produced in 5-day-old carboxymethyl cellulose (CMC) cultures. ND, not detected; WT, *F. graminearum* wild-type strain Z-3639; Δ*abaA*, *abaA* deletion mutant; AbaAc, Δ*abaA-*derived strain complemented with *abaA* of *F. graminearum*; AnAbaAc, Δ*abaA-*derived strain complemented with *abaA* of *A. nidulans* (*AnAbaA*).

For inter- and intra-species genetic complementations, the *abaA* ORF fused with *gfp* and the *AnabaA* ORF of *A. nidulans* driven by the *abaA* native promoter of *F. graminearum* were introduced into an *abaA* deletion mutant resulting in AbaAc and AnAbaAc strains, respectively. All of the complemented mutants were confirmed with Southern hybridizations (Figure S1). Conidium production of AbaAc complemented strains demonstrated that the AbaA-GFP fully functional for conidiogenesis (Figure 4). The interspecies-complemented AnAbaAc strains also completely restored defective conidiogenesis of the *abaA* deletion mutants.

### AbaA-GFP localization

**Figure 5:**
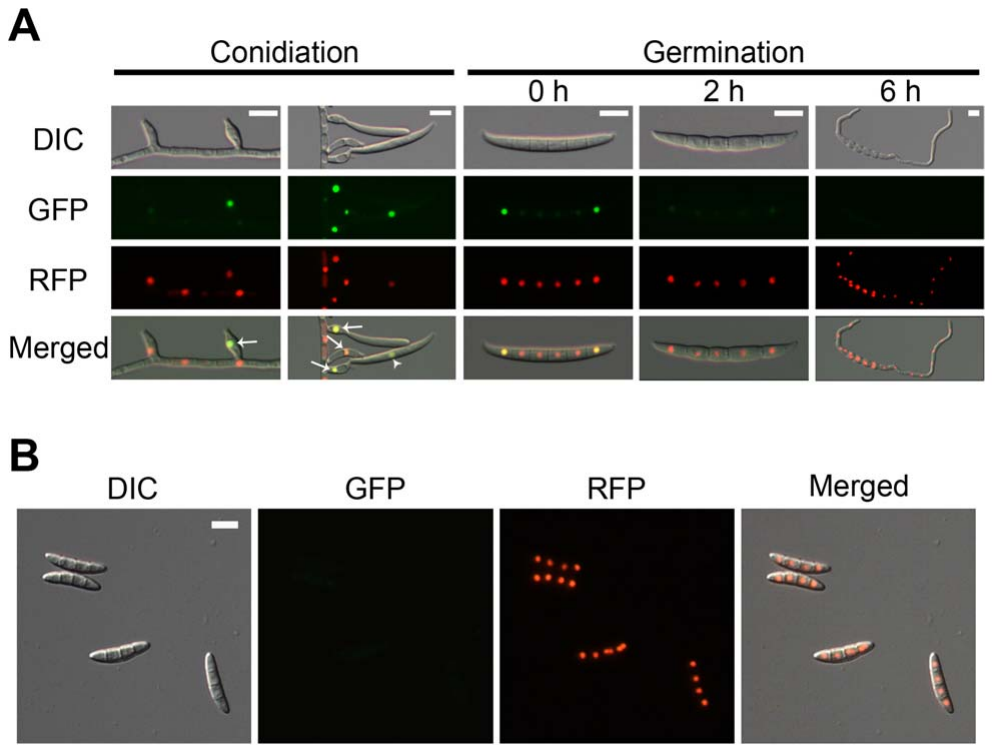
Cellular localization of AbaA. AbaA was fused with green fluorescent protein (GFP), and histone H1 was fused with red fluorescent protein (RFP). (A) The GFP signals were highly fluorescent in the phialides (indicated with arrows) and maturing conidia (indicated with arrow heads). The GFP signals became blurred after germination. (B) The GFP signals were undetectable in mature ascospores. Scale bar = 10 µm.

To examine AbaA localization, the *abaA-gfp* fusion construct under the control of its native promoter was transformed into the *abaA* deletion mutant for genetic complementation (Figure S1A). To confirm nuclear localization of AbaA-GFP, the AbaAcr (Δ*abaA::abaA-gfp-hyg*; *hH1-rfp-gen*) strain was generated by an outcross between the mat1r [18] and the AbaAc strains. AbaA-GFP in the AbaAcr strain colocalized with hH1-RFP (Figure 5). The GFP signals were highly fluorescent in the nuclei of phialides and mature conidia. In mature conidia, AbaA-GFP highly accumulated in the nuclei of terminal cells. However, the GFP signals became blurred after the conidia began to germinate and were undetectable 6 h after germination (Figure 5A). The GFP signal was undetectable in mature ascospores (Figure 5B).

### Overexpression of *abaA*

**Figure 6:**
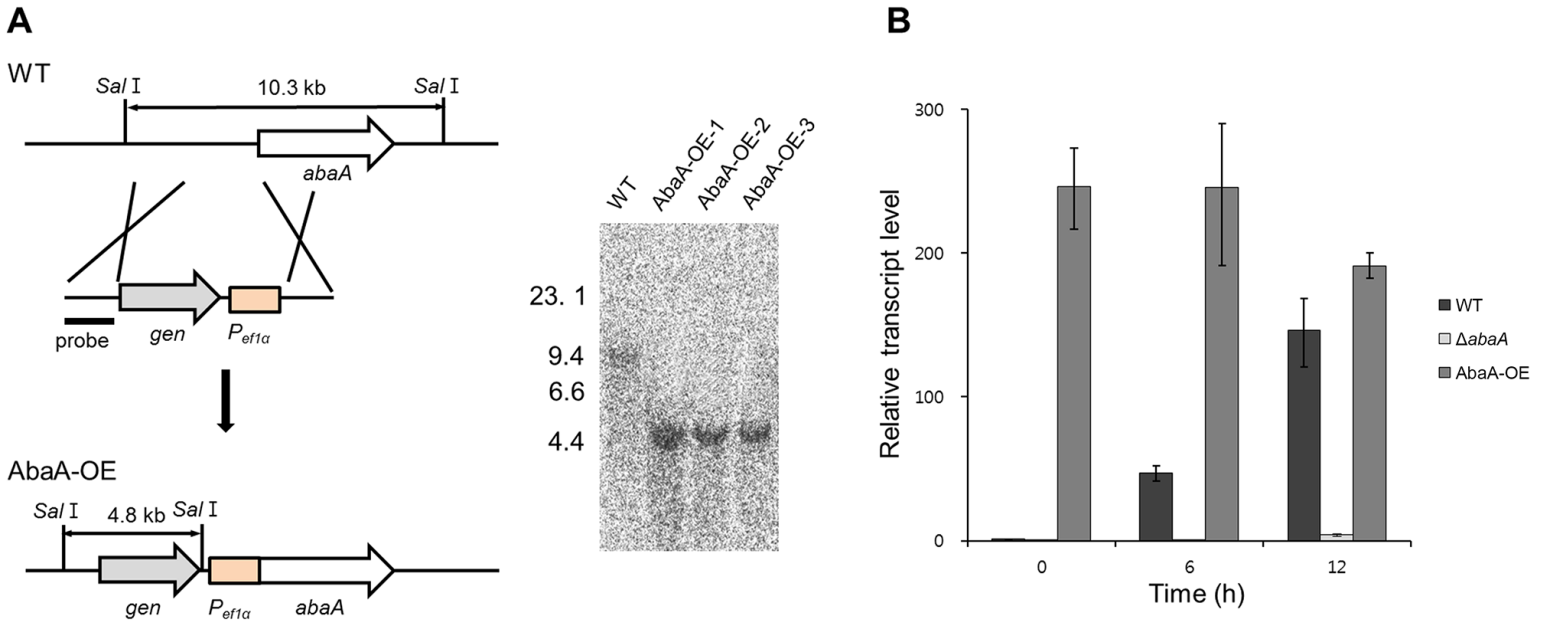
Overexpression of *abaA*. (A) The *abaA* promoter was replaced with the *ef1α* promoter. The left and right panels show the strategy of AbaA-OE strain construction and Southern hybridization, respectively. The sizes of DNA standards (kb) are indicated on the left of the blot. (B) Relative transcript accumulation of *abaA* in wild-type, *abaA*-deleted, and *abaA*-overexpressed strains. The transcript level of *abaA* was analyzed by quantitative real time-PCR (qRT-PCR) during the conidium induction stage. WT, *F. graminearum* wild-type strain Z-3639; Δ*abaA*, *abaA* deletion mutant; AbaA-OE, a transgenic strain with the *abaA* promoter replaced with the *ef1α* promoter.

We also generated *abaA* overexpression strains (AbaA-OE) in which *abaA* expression was under the control of a strong promoter, the *ef1α* promoter of *F. verticillioides*. Southern hybridization was performed to confirm the expected genetic manipulation (Figure 6A). The *abaA* transcript level in wild-type and AbaA-OE strains was determined by qRT-PCR. In the wild-type strain, the *abaA* expression level was significantly upregulated at 6 h after conidium induction, which increased until 12 h. In the AbaA-OE strain, the transcript level was constitutively upregulated during conidiogenesis compared to the wild-type strain (Figure 6B). *abaA* deletion completely abolished *abaA* transcript accumulation.

**Figure 7:**
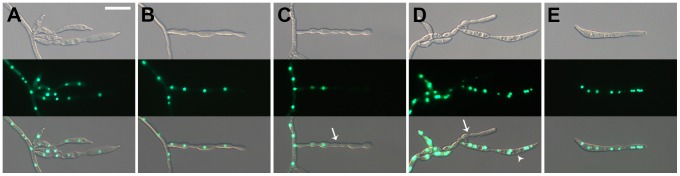
Morphology of conidiophores of AbaA-OE strains. Pictures were taken 1 to 3 days after conidium induction from carboxymethyl cellulose (CMC) medium. Multiple phialides were produced from a single hyphal compartment (A). Some phialides resembled abacus-like structures, and some cells near the end did not contain detectable nuclei (B–D). (E) Conidia produced from AbaA-OE strains (E). Cells that did not contain detectable nuclei and mature conidia resembling abacus-like structures are indicated by white arrows and arrow heads, respectively. Scale bar = 20 µm.

*abaA* overexpression mutants produced increased amounts of aerial mycelia and total biomass compared to the other strains (Table 2 and Figure 2A). Number of perithecia and total trichothecene (DON + 15-AcDON) production were reduced in AbaA-OE strains compared to the wild-type strain (Table 2 and Figure 2B); furthermore, ascospore maturation was also delayed by one day (Figure 2C). The virulence of AbaA-OE mutants was not significantly different from the wild-type strain (Figure 2D). Moreover, AbaA-OE strains produced fewer numbers of conidia compared to the wild-type strain (0.1-fold), with extremely abnormal morphology (Figure 7). Multiple phialides were induced from a single hyphal cell (Figure 7A). Many phialides of the AbaA-OE strains resembled abacus-like structures, and some cells near the end did not contain detectable nuclei (Figure 7C and D). Conidia produced from the AbaA-OE strains were multi-nucleated and were likely produced by abscission of abacus-like structures (Figure 7D and E). However, hyphal morphology and septation and nuclei distribution patterns were not markedly different between the wild type and AbaA-OE strains (Figure S2).

**Figure 8:**
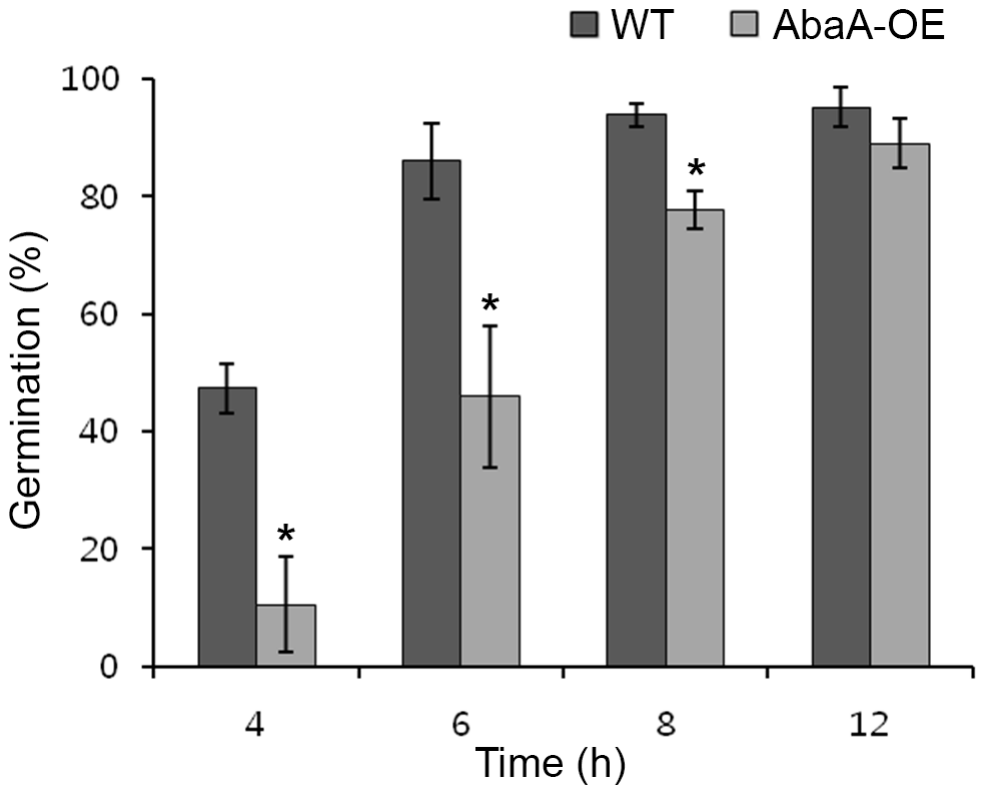
Germination rate of conidia. One ml of conidium suspension of each strain was incubated in 10 ml of complete medium (CM) at 25°C on a rotary shaker (150 rpm). One hundred spores were observed in each examination with light microscopy, and the number of conidia that germinated was counted. All data were obtained from three biological replicates. Asterisks indicate data that significantly differed (*p*<0.05) based on Tukey's test. WT, *F. graminearum* wild-type strain Z-3639; AbaA-OE, a transgenic strain with the *abaA* promoter replaced with the *ef1α* promoter.

Overexpression of *abaA* led to a decrease in the germination rate. In the wild-type strain, 47% and 86% of conidia germinated 4 h and 6 h after incubation, respectively. In AbaA-OE mutants, approximately 10% of conidia germinated 4 h after incubation, and 46% of conidia germinated within 6 h of incubation in CM (Figure 8). After incubation for 12 h, there was no significant difference in the germination rates between wild-type and *abaA* overexpression mutant strains.

### Genetic relationship between *abaA* and *wetA*

**Figure 9:**
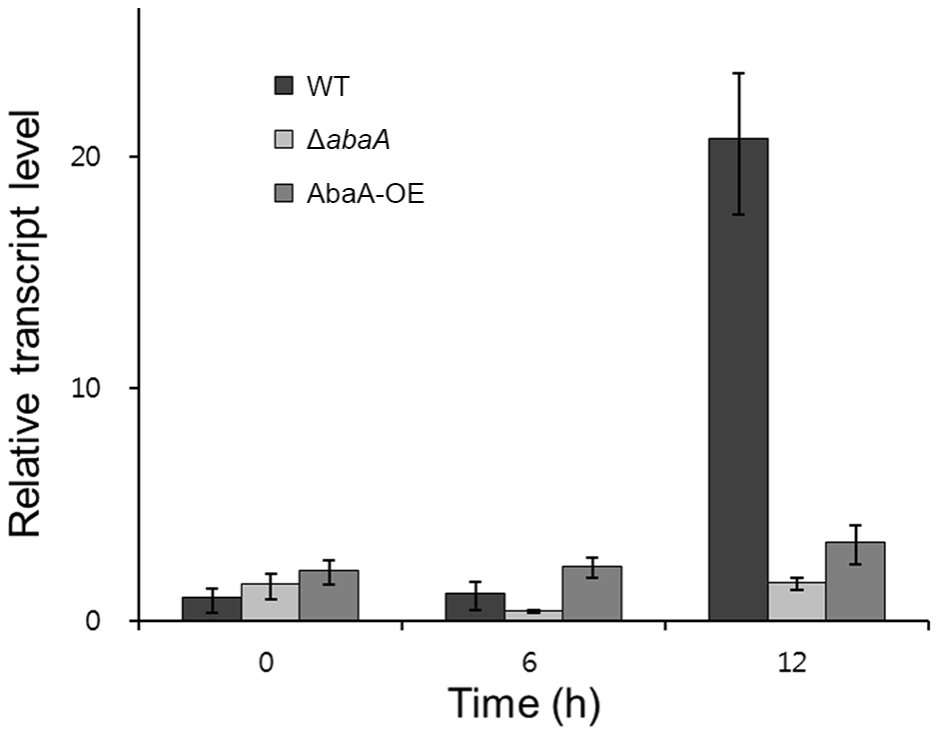
Relative transcript accumulation of *wetA*. The transcript levels of *wetA* (FGSG_17727) were analyzed by quantitative real time-PCR (qRT-PCR) during the conidium induction stage in the wild-type, *abaA* deletion mutant, and *abaA* overexpression mutant strains. WT, *F. graminearum* wild-type strain Z-3639; Δ*abaA*, *abaA* deletion mutant; AbaA-OE, a transgenic strain with the *abaA* promoter replaced with the *ef1α* promoter.

Since *wetA* is known to be a direct target gene of AbaA in *Aspergilli* [60], [64], the transcript level of *wetA* homolog genes (FGSG_10166, 24% identity) was analyzed in the wild-type and *abaA* deletion mutants. In the wild-type strain, the *wetA* expression level was significantly upregulated at 12 h after conidium induction (Figure 9). However, the transcript level of *wetA* was similarly or constitutively downregulated during conidiogenesis in *abaA* deletion mutants compared to the wild-type strain. In *abaA* overexpression mutant, transcript level of *wetA* was slightly increased compared to the *abaA* deletion mutants.

### Determination of AbaA-dependent pathways through transcriptomic analysis

To understand the pathways regulated by AbaA, we obtained and analyzed the transcriptional profiles from RNA-sequencing data using two strains. We compared the Δ*abaA* mutants with the *F. graminearum* wild-type strain at three different time points (0 h, 6 h, and 12 h after induction of conidiogenesis). To identify the AbaA-regulated genes, differentially expressed genes (DEGs) were selected from 2.0-fold changes of RPKM values [59]. Based on this criterion, 3303 and 3579 genes were identified 6 h after and 12 h after induction of conidiogenesis, respectively. In addition, 946 genes were expressed in an AbaA-dependent manner both 6 h and 12 h after induction of conidiogenesis.

For pathway analysis data downloaded from the *F. graminearum* KEGG database, 22 out of 101 pathways had at least two DEGs either 6 h or 12 h after induction of conidiogenesis. Most genes involved in these 22 pathways were upregulated (Table S2). In the beginning of conidiogenesis (6 h after induction), many genes involved in alanine, aspartate, and glutamate metabolism; glycolysis/gluconeogenesis and fatty acid pathways; and vitamin B6 metabolism were upregulated (Table S2). All annotated genes (7 out of 7) involved in fatty acid pathways were upregulated. At the end of conidiogenesis (12 h after induction), 9 and 7 genes involved in the cell cycle and DNA replication were upregulated, respectively (Table S2).

### RNA sequencing-based expression patterns of core genes involved in conidiogenesis

**Figure 10:**
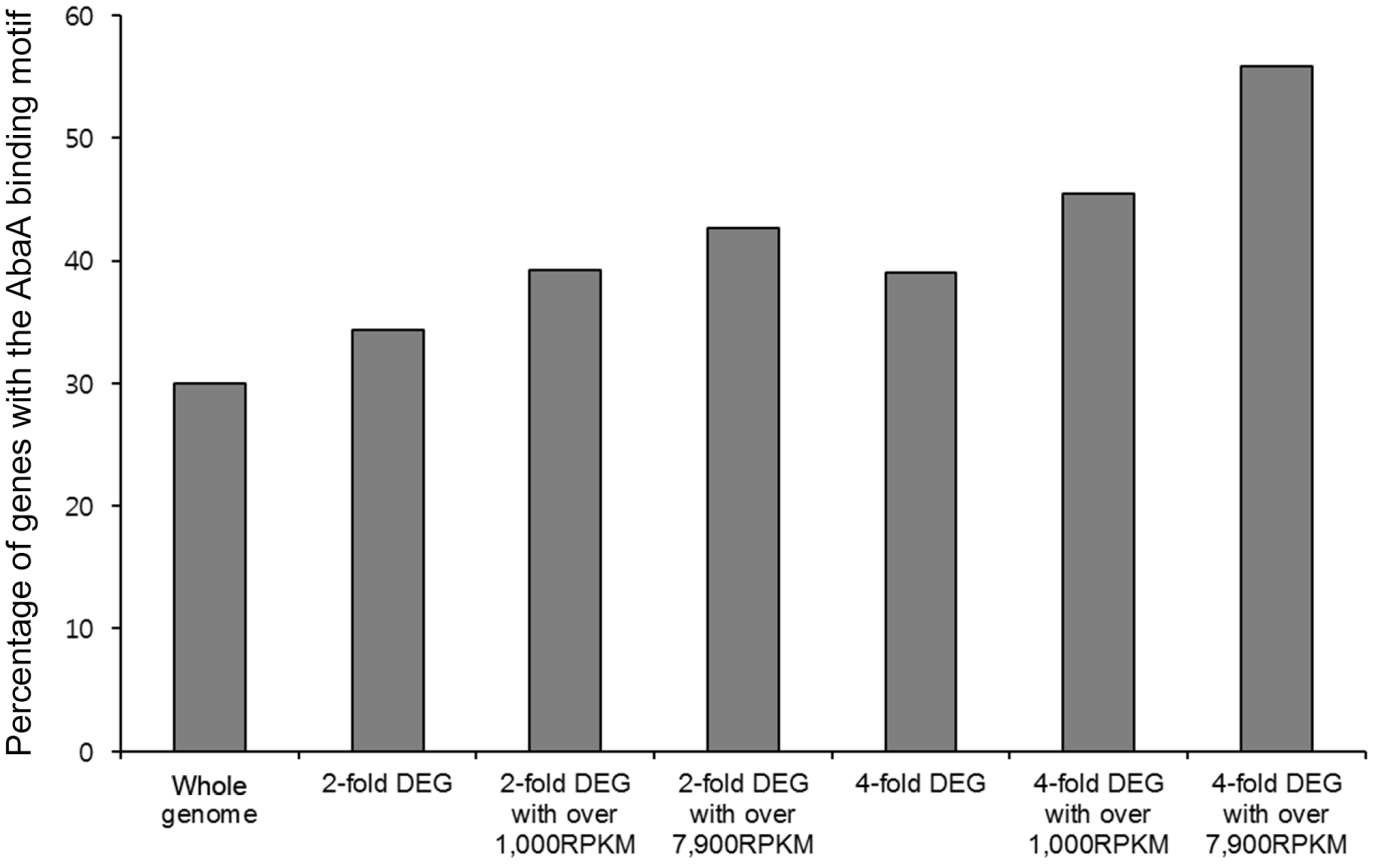
Percentage of DEGs among AbaA binding motif-containing genes. All information on *F. graminearum* sequences and annotation was obtained from the *F. graminearum* Database (http://mips.helmholtz-muenchen.de/genre/proj/FGDB/) [51]. From this genome sequence and gene annotation, 500-bp upstream sequences were collected from all predicted genes and surveyed for finding CATTCY motifs in these regions [60]. DEGs between *F. graminearum* wild-type strain Z-3639 and *abaA* deletion mutant were compared with genes having CATTCY motifs within 500-bp upstream sequences to calculate percentages of DEGs among CATTCY motifs -containing genes. DEG, differentially expressed genes; RPKM, reads per kilobase of exon per million.

Due to successful complementation of *A. nidulans* AbaA in the Δ*abaA* mutant, *F. graminearum* AbaA supposed to have shared features such as a conserved binding motif (CATTCY). Thus, we surveyed this binding motif in the upstream regions (−500 to −1 bp) of genes involved in conidiogenesis and AbaA-regulated genes. Five out of eight genes possibly involved as key regulators of condiogenesis have at least one motif in the surveyed upstream (promoter) regions (Table S3). In the promoter regions of *abaA* and *wetA*, multiple binding sites were observed, which is similar to results found with *A. nidulans* and *A. fumigatus* [60], [64]. From the genome-wide survey of this binding motif, 30% (4156/13820) of genes were found to have this motif. Interestingly, genes that were more tightly regulated by AbaA as well as more abundantly expressed were more likely to have this motif. In the case of AbaA-regulated genes both 6 h and 12 h after induction of conidiogenesis, we found that 55.8% (29/52) of the genes had at least a 4-fold change and were greater than 7900 RPKM (an average expression per gene in this RNA-sequencing analysis) (Figure 10). With the same criteria except for having at least a 2-fold change, 42.7% (91/213) of genes had this motif (Table S4). From this analysis, we found three putative AbaA target genes that were already known to be required for conidiation in *F. graminearum*: FgAxl2 (FGSG_10237), one kinase (FGSG_16988), and one transcription factor (GzHOME005; FGSG_16777) [44], [45], [65].

Due to reports on cell cycle control via *A. nidulans* AbaA, we monitored the expression patterns of 53 genes involved in cell cycle pathways obtained from the KEGG database. We observed that 14 out of 53 genes were upregulated with a 1.7-fold change 12 h after induction of conidiogenesis (Table S5).

## Discussion

In *A. nidulans*, conidiation begins with cessation of vegetative growth and with subsequent formation of the footcell and the stalk of the hyphal cell. After stalk elongation to some extent, a globular structure called the vesicle is formed at the stalk tip and then metulae are produced by multipolar budding from the vesicle. Two apical buds emerge from each mature metulae to become phialides. Finally, single-celled conidia are basipetally produced from the mature phialide [35]. Like this, morphological transformations leading to a conidiophore include several steps of cessation of cell elongation/division and specialized cell differentiations in *A. nidulans*. Therefore, these processes require precise control of cell division and differentiation at the molecular level both in terms of time and location. Decades of genetic and biochemical studies have constructed genetic networks and have revealed the mechanistic roles of the factors required for conidiogenesis in the model filamentous fungus *A. nidulans* [35], [36], [61], [66], [67].

Compared to conidiation processes in *A. nidulans*, *F. graminearum* has a much simpler conidiogenesis system. *F. graminearum* directly produces a conidiogenous cell, i.e., a phialide, from the hyphae and continuously generates multiseptate conidia [1], [47]. Since conidia are responsible for disease propagation and conidiation is a unique characteristic of fungal species, it has been hypothesized that conidiogenesis-related genes might be novel targets for disease control [6]. Therefore, the aim of this study was to elucidate the molecular mechanisms underlying conidiogenesis in *F. graminearum* based on the framework provided in the conidiation study in *A. nidulans*.

In this study, we first found and characterized the functions of the *abaA* ortholog gene, which is known to be absent in *F. graminearum*. AbaA is specifically required for morphogenesis and differentiation of phialides in *F. graminearum*, similar to that in *A. nidulans*. The interspecies complementation assay additionally demonstrated that the molecular mechanisms of AbaA activity are also conserved in *F. graminearum* as they are in *A. nidulans*. AbaA predominantly localized to the nuclei of phialides and terminal cells of mature conidia. Overexpression and constitutive expression of *abaA* resulted in pleiotropic defects such as impaired sexual and asexual development and decreased conidium germination rate and trichothecene production. Furthermore, three putative AbaA target genes that were already known to be required for conidiation in *F. graminearum* were found [44], [45], [65].

The AbaA of *F. graminearum* has some conserved functions as those of other fungi in conidiogenesis. Abacus mutants (knockout mutants of *abaA*) of *A. nidulans* produce nearly normal conidiophores bearing stalk, vesicle, and metulae. However, abacus-like structures with properties of metulae are continuously formed from the metulae instead of normal phialides [30], [38], [39]. Similarly, *abaA*-null mutants of *A. fumigatus* and *Penicillium marneffei* also show defects similar to those of *A. nidulans* [64], [68]. *abaA* deletion mutants of *F. graminearum* produced impaired phialides, which failed to make conidia but instead made thin hyphae (Figure 3). Even though conidiophore structures of *F. graminearum*, *Aspergilli*, and *P. marneffei* are different from each other, AbaA has conserved roles in phialide formation and functions in these fungal species.

Another distinct function of AbaA in *F. graminearum* conidiogenesis is as a regulator for cell division in conidia. Conidium maturation in *F. graminearum* requires specific cell differentiation processes, which inhibit filamentous growth but maintain cell elongation with appropriate septum formation and nuclear division inside of the conidium. *abaA* deletion resulted in failure of conidium formation from the phialide (Figure 3), and *abaA* overexpression mutants delayed conidial germination in *F. graminearum* (Figure 8). Moreover, AbaA proteins were highly accumulated in mature conidia and the terminal cells of conidia (Figure 5). The terminal cells of conidia tend to preferentially germinate compared to intercalary cells [69]. In addition, AbaA is well known as a cell cycle regulator in *A. nidulans* and *P. marneffei*, and an *abaA* mutation in *A. nidulans* caused disruption of G1 arrest of conidium initial cells [68], [70]. Furthermore, AbaA can regulate the cell cycle in the late stage rather than in the early stage of conidiogenesis. Taken together, we suggest that AbaA is required for maturation and dormancy of conidia in *F. graminearum* through cell cycle regulation.

The presence of the AbaA ortholog gives new insights into the genetics of asexual spore formation in *F. graminearum*. A previous comparative genomic analysis of conidiation-related regulators showed that the *F. graminearum* genome does not have AbaA and BrlA but does have FlbC, FlbD, FluG, and WetA orthologs [61]. However, we found that the AbaA ortholog exists and that the AbaA-WetA pathway is also conserved in *F. graminearum*. In the case of BrlA, we utilized the previously generated *F. graminearum* Transcription Factor Phenotype Database [45] and found that a homolog of BrlA (GzBrlA, 17% identity) is not related to conidiation in *F. graminearum*. This result is consistent with morphological differences of conidiophores between *A. nidulans* and *F. graminearum*. The *A. nidulans* conidiophore bears vesicles whereas that of *F. graminearum* does not; and BrlA is a key transcription factor required for vesicle formation [37]. Moreover, deletion of the homologous genes of FlbC (GzC2H2047, 32% identity) and FlbD (GzFlbD, 35% identity) resulted in reduced and abolished conidiation in *F. graminearum*, respectively [45], suggesting that some UDAs might have conserved roles for conidiation in both *A. nidulans* and *F. graminearum*.

Some unexpected results of this study also suggest that there are considerable differences between *A. nidulans* and *F. graminearum* in mechanism of action of AbaA. In *A. nidulans* phialide cells, *An*AbaA both activates *velB* and *vosA* and resulting gene products represses *brlA* expression [66]. Since BrlA is a direct upstream regulator for *abaA* in BrlA-AbaA-WetA pathway, the amount of *abaA* mRNA started to be reduced 24 h after conidia induction and mostly disappear in conidia [71]. However, AbaA was highly accumulated in conidium terminal cells in *F. graminearum* indicating that additional functions of AbaA exist in *F. graminearum* conidia as mentioned above. Similarly *abaA* overexpression results in opposite phenotypic changes in *A. nidulans* and *F. graminearum*. Overexpression of *AnabaA* causes growth cessation [39] whereas AbaA-OE mutants showed excessive production of aerial mycelia and biomass. These differences seem to be firstly derived from lack of conserved genes between two fungi e.g. *brlA* and *vosA*. In addition, transcriptional rewiring might be the reason for these phenomena since orthologs of transcriptional factors can participate in different transcriptional regulations because of variable *Cis*-regulatory elements [72], [73]. Our results from promoter analysis also support this hypothesis.

Although there are distinct morphological differences between conidiophores of *A. nidulans* and *F. graminearum*, AbaA has conserved roles for phialide formation and function in both fungi. As a key cell cycle regulator during conidiation, AbaA is additionally required for conidium maturation and dormancy in *F. graminearum*. Utilizing our previous phenome database and our current results, we suggest that homologs of FlbC and FlbD and the AbaA-WetA pathway have conserved roles for conidiation in *F. graminearum*. Future work will focus on identifying other putative regulators related to conidiation in *F. graminearum* based on *A. nidulans* studies, allowing us to construct genetic networks underlying conidiogenesis in this fungus.

## Supporting Information

Figure S1 **Complementation of** ***abaA*** **.** Intra- (A) and interspecies (B) complementation of *abaA* in the *abaA* deletion mutant. Δ*abaA*, *abaA* deletion mutant; AbaAc, Δ*abaA-*derived strain complemented with *abaA* of *F. graminearum*; AnAbaAc, Δ*abaA-*derived strain complemented with *abaA* of *A. nidulans* (*AnabaA*); *gen*, geneticin resistance gene cassette; *hyg*, hygromycin B resistance gene cassette. The sizes of DNA standards (kb) are indicated on the left of the blot.
(TIF)

Figure S2 **Mycelial morphology of** ***F. graminearum*** **strains.** Differential interference contrast (DIC) and fluorescent microscopic observations were conducted 1 day after inoculation in complete medium (CM). Chitin accumulation in hyphae was visualized with Calcofluor white (CFW) staining. WT, hH1-GFP strain; Δ*abaA*, Δ*abaA*-g strain; AbaA-OE, AbaA-OE-g strain. Scale bar = 50 µm.
(TIF)

Table S1 **Primers used in this study.**
(PDF)

Table S2 **Distribution of AbaA-dependent genes in the pathway.**
(XLS)

Table S3 **RNA sequencing-based expression patterns of core genes involved in conidiogenesis in the Δ** ***abaA*** **mutant strain.**
(XLS)

Table S4 **Differentially expressed AbaA-dependent genes (>4-fold).**
(XLS)

Table S5 **Expression patterns of genes involved in cell cycle pathways.**
(XLS)

## References

[1] Leslie JF, Summerell BA (2006) The *Fusarium* laboratory manual. Ames, IA: Blackwell Pub.

[2] Desjardins AE, Proctor RH (2007) Molecular biology of *Fusarium* mycotoxins. Int J Food Microbiol 119: 47–50. 17707105 10.1016/j.ijfoodmicro.2007.07.024

[3] Marasas WFO, Nelson PE, Toussoun TA (1984) Toxigenic *Fusarium* species, identity and mycotoxicology. University Park, PA: The Pennsylvania State University Press.

[4] Trail F, Xu H, Loranger R, Gadoury D (2002) Physiological and environmental aspects of ascospore discharge in *Gibberella zeae* (anamorph *Fusarium graminearum*). Mycologia 94: 181–189. 21156487

[5] Sutton JC (1982) Epidemiology of wheat head blight and maize ear rot caused by *Fusarium graminearum* . Can J Plant Pathol 4: 195–209.

[6] Guenther JC, Trail F (2005) The development and differentiation of *Gibberella zeae* (anamorph: *Fusarium graminearum*) during colonization of wheat. Mycologia 97: 229–237. 16389974 10.3852/mycologia.97.1.229

[7] Goh YK, Daida P, Vujanovic V (2009) Effects of abiotic factors and biocontrol agents on chlamydospore formation in *Fusarium graminearum* and *Fusarium sporotrichioides* . Biocontrol Sci Technol 19: 151–167.

[8] Nyvall RF (1970) Chlamydospores of *Fusarium roseum* ‘Graminearum’ as survival structures. Phytopathology 60: 1175–1177.

[9] Baldwin TK, Urban M, Brown N, Hammond-Kosack KE (2010) A role for topoisomerase I in *Fusarium graminearum* and *F*. *culmorum* pathogenesis and sporulation. Mol Plant-Microbe Interact 23: 566–577. 20367465 10.1094/MPMI-23-5-0566

[10] Bluhm BH, Zhao X, Flaherty JE, Xu J-R, Dunkle LD (2007) *RAS2* regulates growth and pathogenesis in *Fusarium graminearum* . Mol Plant-Microbe Interact 20: 627–636. 17555271 10.1094/MPMI-20-6-0627

[11] Ding S, Mehrabi R, Koten C, Kang Z, Wei Y, et al (2009) Transducin beta-like gene *FTL1* Is essential for pathogenesis in *Fusarium graminearum* . Eukaryot Cell 8: 867–876. 19377037 10.1128/EC.00048-09 PMC2698311

[12] Han Y-K, Kim M-D, Lee S-H, Yun S-H, Lee Y-W (2007) A novel F-box protein involved in sexual development and pathogenesis in *Gibberella zeae* . Mol Microbiol 63: 768–779. 17302802 10.1111/j.1365-2958.2006.05557.x

[13] Hou Z, Xue C, Peng Y, Katan T, Kistler HC, et al (2002) A mitogen-activated protein kinase gene (*MGV1*) in *Fusarium graminearum* is required for female fertility, heterokaryon formation, and plant infection. Mol Plant-Microbe Interact 15: 1119–1127. 12423017 10.1094/MPMI.2002.15.11.1119

[14] Jenczmionka NJ, Maier FJ, Lösch AP, Schäfer W (2003) Mating, conidiation and pathogenicity of *Fusarium graminearum*, the main causal agent of the head-blight disease of wheat, are regulated by the MAP kinase gpmk1. Curr Genet 43: 87–95. 12695848 10.1007/s00294-003-0379-2

[15] Li Y, Wang C, Liu W, Wang G, Kang Z, et al (2011) The *HDF1* histone deacetylase gene is important for conidiation, sexual reproduction, and pathogenesis in *Fusarium graminearum* . Mol Plant-Microbe Interact 24: 487–496. 21138346 10.1094/MPMI-10-10-0233

[16] Lysøe E, Pasquali M, Breakspear A, Kistler HC (2011) The transcription factor FgStuAp influences spore development, pathogenicity, and secondary metabolism in *Fusarium graminearum* . Mol Plant-Microbe Interact 24: 54–67. 20879840 10.1094/MPMI-03-10-0075

[17] Rittenour WR, Harris SD (2008) Characterization of *Fusarium graminearum* Mes1 reveals roles in cell-surface organization and virulence. Fungal Genet Biol 45: 933–946. 18339563 10.1016/j.fgb.2008.01.007

[18] Son H, Lee J, Park AR, Lee Y-W (2011) ATP citrate lyase is required for normal sexual and asexual development in *Gibberella zeae* . Fungal Genet Biol 48: 408–417. 21237280 10.1016/j.fgb.2011.01.002

[19] Urban M, Mott E, Farley T, Hammond-Kosack K (2003) The *Fusarium graminearum MAP1* gene is essential for pathogenicity and development of perithecia. Mol Plant Pathol 4: 347–359. 20569395 10.1046/j.1364-3703.2003.00183.x

[20] Yu H-Y, Seo J-A, Kim J-E, Han K-H, Shim W-B, et al (2008) Functional analyses of heterotrimeric G protein Gα and Gβ subunits in *Gibberella zeae* . Microbiology-(UK) 154: 392. 10.1099/mic.0.2007/012260-0 PMC2885625 18227243

[21] Zhou X, Heyer C, Choi Y-E, Mehrabi R, Xu J-R (2010) The *CID1* cyclin C-like gene is important for plant infection in *Fusarium graminearum* . Fungal Genet Biol 47: 143–151. 19909822 10.1016/j.fgb.2009.11.001

[22] Lin Y, Son H, Lee J, Min K, Choi GJ, et al (2011) A putative transcription factor MYT1 is required for female fertility in the ascomycete *Gibberella zeae* . PLoS One 6: e25586. 21984921 10.1371/journal.pone.0025586 PMC3184970

[23] Lin Y, Son H, Min K, Lee J, Choi GJ, et al (2012) A putative transcription factor MYT2 regulates perithecium size in the ascomycete *Gibberella zeae* . PLoS One 7: e37859. 22649560 10.1371/journal.pone.0037859 PMC3359310

[24] Wang Y, Liu W, Hou Z, Wang C, Zhou X, et al (2011) A novel transcriptional factor important for pathogenesis and ascosporogenesis in *Fusarium graminearum* . Mol Plant-Microbe Interact 24: 118–128. 20795857 10.1094/MPMI-06-10-0129

[25] Son H, Min K, Lee J, Choi GJ, Kim J-C, et al (2012) Mitochondrial carnitine-dependent acetyl coenzyme A transport is required for normal sexual and asexual development of the ascomycete *Gibberella zeae* . Eukaryot Cell 11: 1143–1153. 22798392 10.1128/EC.00104-12 PMC3445975

[26] Son H, Min K, Lee J, Choi GJ, Kim J-C, et al (2012) Differential roles of pyruvate decarboxylase in aerial and embedded mycelia of the ascomycete *Gibberella zeae* . FEMS Microbiol Lett 329: 123–130. 22276936 10.1111/j.1574-6968.2012.02511.x

[27] Son H, Lee J, Lee Y-W (2013) A novel gene, *GEA1*, is required for ascus cell wall development in the ascomycete fungus, *Fusarium graminearum* . Microbiology-(UK) 159: 1077–1085. 10.1099/mic.0.064287-0 23619001

[28] Lee S-H, Lee J, Lee S, Park E-H, Kim K-W, et al (2009) *GzSNF1* is required for normal sexual and asexual development in the ascomycete *Gibberella zeae* . Eukaryot Cell 8: 116–127. 19028993 10.1128/EC.00176-08 PMC2620747

[29] Zheng W, Zhao X, Xie Q, Huang Q, Zhang C, et al (2012) A conserved homeobox transcription factor Htf1 is required for phialide development and conidiogenesis in *Fusarium* Species. PLoS One 7: e45432. 23029006 10.1371/journal.pone.0045432 PMC3448628

[30] Clutterbuck AJ (1969) A mutational analysis of conidial development in *Aspergillus nidulans* . Genetics 63: 317. 5366214 10.1093/genetics/63.2.317 PMC1212347

[31] Garnjobst L, Tatum EL (1967) A survey of new morphological mutants in *Neurospora crassa* . Genetics 57: 579. 5583730 10.1093/genetics/57.3.579 PMC1211750

[32] Martinelli SD, Clutterbuck AJ (1971) A quantitative survey of conidiation mutants in *Aspergillus nidulans* . Microbiology-(UK) 69: 261. 10.1099/00221287-69-2-261 4947820

[33] Miller BL (1990) The developmental genetics of asexual reproduction in *Aspergillus nidulans* . Semin Dev Biol 1: 207–219.

[34] Springer ML (1993) Genetic control of fungal differentiation: The three sporulation pathways of *Neurospora crassa* . BioEssays 15: 365–374. 8357339 10.1002/bies.950150602

[35] Etxebeste O, Garzia A, Espeso EA, Ugalde U (2010) *Aspergillus nidulans* asexual development: making the most of cellular modules. Trends Microbiol 18: 569–576. 21035346 10.1016/j.tim.2010.09.007

[36] Adams TH, Wieser JK, Yu J-H (1998) Asexual sporulation in *Aspergillus nidulans* . Microbiol Mol Biol Rev 62: 35–54. 9529886 10.1128/mmbr.62.1.35-54.1998 PMC98905

[37] Adams TH, Boylan MT, Timberlake WE (1988) *brlA* is necessary and sufficient to direct conidiophore development in *Aspergillus nidulans* . Cell 54: 353–362. 3293800 10.1016/0092-8674(88)90198-5

[38] Sewall TC, Mims CW, Timberlake WE (1990) *abaA* controls phialide differentiation in *Aspergillus nidulans* . Plant Cell 2: 731–739. 2152124 10.1105/tpc.2.8.731 PMC159926

[39] Mirabito PM, Adams TH, Timberlake WE (1989) Interactions of three sequentially expressed genes control temporal and spatial specificity in *Aspergillus* development. Cell 57: 859–868. 2655931 10.1016/0092-8674(89)90800-3

[40] Marshall MA, Timberlake WE (1991) *Aspergillus nidulans wetA* activates spore-specific gene expression. Mol Cell Biol 11: 55–62. 1986246 10.1128/mcb.11.1.55-62.1991 PMC359587

[41] Bayram Ö, Krappmann S, Ni M, Bok JW, Helmstaedt K, et al (2008) VelB/VeA/LaeA complex coordinates light signal with fungal development and secondary metabolism. Science 320: 1504–1506. 18556559 10.1126/science.1155888

[42] Jeon J, Park S-Y, Chi M-H, Choi J, Park J, et al (2007) Genome-wide functional analysis of pathogenicity genes in the rice blast fungus. Nat Genet 39: 561–565. 17353894 10.1038/ng2002

[43] Shi Z, Leung H (1995) Genetic analysis of sporulation in *Magnaporthe grisea* by chemical and insertional mutagenesis. Mol Plant-Microbe Interact 8: 949–959.

[44] Wang C, Zhang S, Hou R, Zhao Z, Zheng Q, et al (2011) Functional analysis of the kinome of the wheat scab fungus *Fusarium graminearum* . PLoS Pathog 7: e1002460. 22216007 10.1371/journal.ppat.1002460 PMC3245316

[45] Son H, Seo Y-S, Min K, Park AR, Lee J, et al (2011) A phenome-based functional analysis of transcription factors in the cereal head blight fungus, *Fusarium graminearum* . PLoS Pathog 7: e1002310. 22028654 10.1371/journal.ppat.1002310 PMC3197617

[46] O'Donnell K, Kistler HC, Tacke BK, Casper HH (2000) Gene genealogies reveal global phylogeographic structure and reproductive isolation among lineages of *Fusarium graminearum*, the fungus causing wheat scab. Proc Natl Acad Sci USA 97: 7905–7910. 10869425 10.1073/pnas.130193297 PMC16643

[47] Harris SD (2005) Morphogenesis in germinating *Fusarium graminearum* macroconidia. Mycologia 97: 880–887. 16457357 10.3852/mycologia.97.4.880

[48] Cappellini RA, Peterson JL (1965) Macroconidium formation in submerged cultures by a non-sporulating strain of *Gibberella zeae* . Mycologia 57: 962–966.

[49] Gardiner DM, Kazan K, Manners JM (2009) Novel genes of *Fusarium graminearum* that negatively regulate deoxynivalenol production and virulence. Mol Plant-Microbe Interact 22: 1588–1600. 19888824 10.1094/MPMI-22-12-1588

[50] Sambrook J, Russell DW (2001) Molecular cloning: a laboratory manual, 2nd ed. Cold Spring Harbor, NY: Cold Spring Harbor Laboratory Press.

[51] Wong P, Walter M, Lee W, Mannhaupt G, Münsterkötter M, et al (2011) FGDB: revisiting the genome annotation of the plant pathogen *Fusarium graminearum* . Nucleic Acids Res 39: D637–D639. 21051345 10.1093/nar/gkq1016 PMC3013644

[52] Yu JH, Hamari Z, Han KH, Seo JA, Reyes-Dominguez Y, et al (2004) Double-joint PCR: a PCR-based molecular tool for gene manipulations in filamentous fungi. Fungal Genet Biol 41: 973–981. 15465386 10.1016/j.fgb.2004.08.001

[53] Hong S-Y, So J, Lee J, Min K, Son H, et al (2010) Functional analyses of two syntaxin-like SNARE genes, *GzSYN1* and *GzSYN2*, in the ascomycete *Gibberella zeae* . Fungal Genet Biol 47: 364–372. 20102747 10.1016/j.fgb.2010.01.005

[54] Horwitz BA, Sharon A, Lu SW, Ritter V, Sandrock TM, et al (1999) A G protein alpha subunit from *Cochliobolus heterostrophus* involved in mating and appressorium formation. Fungal Genet Biol 26: 19–32. 10072317 10.1006/fgbi.1998.1094

[55] Kim J-E, Han K-H, Jin J, Kim H, Kim J-C, et al (2005) Putative polyketide synthase and laccase genes for biosynthesis of aurofusarin in *Gibberella zeae* . Appl Environ Microbiol 71: 1701–1708. 15811992 10.1128/AEM.71.4.1701-1708.2005 PMC1082506

[56] Lee S, Son H, Lee J, Min K, Choi KJ, et al (2011) Functional analyses of two acetyl coenzyme A synthetases in the ascomycete *Gibberella zeae* . Eukaryot Cell 10: 1043–1052. 21666077 10.1128/EC.05071-11 PMC3165453

[57] Livak KJ, Schmittgen TD (2001) Analysis of relative gene expression data using real-time quantitative PCR and the 2^−ΔΔ*C*T^ method. Methods 25: 402–408. 11846609 10.1006/meth.2001.1262

[58] Son H, Lee J, Lee Y-W (2012) Mannitol induces the conversion of conidia to chlamydospore-like structures that confer enhanced tolerance to heat, drought, and UV in *Gibberella zeae* . Microbiol Res 167: 608–615. 22580127 10.1016/j.micres.2012.04.001

[59] Mortazavi A, Williams BA, McCue K, Schaeffer L, Wold B (2008) Mapping and quantifying mammalian transcriptomes by RNA-Seq. Nat Methods 5: 621–628. 18516045 10.1038/nmeth.1226 PMC13303166

[60] Andrianopoulos A, Timberlake WE (1994) The *Aspergillus nidulans abaA* gene encodes a transcriptional activator that acts as a genetic switch to control development. Mol Cell Biol 14: 2503–2515. 8139553 10.1128/mcb.14.4.2503-2515.1994 PMC358618

[61] Fischer R, Kües U (2006) Asexual sporulation in mycelial fungi. In: Kües U, Fischer R, editors. The Mycota I growth, differentiation and sexuality. Germany: Springer Berlin Heidelberg. pp. 263–292.

[62] Nguyen Ba A, Pogoutse A, Provart N, Moses A (2009) NLStradamus: a simple Hidden Markov Model for nuclear localization signal prediction. BMC Bioinformatics 10: 202. 19563654 10.1186/1471-2105-10-202 PMC2711084

[63] Benson DA, Karsch-Mizrachi I, Clark K, Lipman DJ, Ostell J, et al (2012) GenBank. Nucleic Acids Res 40: D48–D53. 22144687 10.1093/nar/gkr1202 PMC3245039

[64] Tao L, Yu J-H (2011) AbaA and WetA govern distinct stages of *Aspergillus fumigatus* development. Microbiology-(UK) 157: 313–326. 10.1099/mic.0.044271-0 20966095

[65] Si H, Rittenour WR, Xu K, Nicksarlian M, Calvo AM, et al (2012) Morphogenetic and developmental functions of the *Aspergillus nidulans* homologues of the yeast bud site selection proteins Bud4 and Axl2. Mol Microbiol 85: 252–270. 22651396 10.1111/j.1365-2958.2012.08108.x

[66] Park H-S, Yu J-H (2012) Genetic control of asexual sporulation in filamentous fungi. Curr Opin Microbiol 15: 669–677. 23092920 10.1016/j.mib.2012.09.006

[67] Yu J-H (2010) Regulation of development in *Aspergillus nidulans* and *Aspergillus fumigatus* . Mycobiology 38: 229–237. 23956662 10.4489/MYCO.2010.38.4.229 PMC3741515

[68] Borneman AR, Hynes MJ, Andrianopoulos A (2000) The *abaA* homologue of *Penicillium marneffei* participates in two developmental programmes: conidiation and dimorphic growth. Mol Microbiol 38: 1034–1047. 11123677 10.1046/j.1365-2958.2000.02202.x

[69] Seong K-Y, Zhao X, Xu J-R, Güldener U, Kistler HC (2008) Conidial germination in the filamentous fungus *Fusarium graminearum* . Fungal Genet Biol 45: 389–399. 17950638 10.1016/j.fgb.2007.09.002

[70] Ye XS, Lee S-L, Wolkow TD, McGuire S-L, Hamer JE, et al (1999) Interaction between developmental and cell cycle regulators is required for morphogenesis in *Aspergillus nidulans* . EMBO J 18: 6994–7001. 10601021 10.1093/emboj/18.24.6994 PMC1171762

[71] Ni M, Yu J-H (2007) A novel regulator couples sporogenesis and trehalose biogenesis in *Aspergillus nidulans* . PLoS One 2: e970. 17912349 10.1371/journal.pone.0000970 PMC1978537

[72] Tuch BB, Li H, Johnson AD (2008) Evolution of eukaryotic transcription circuits. Science 319: 1797–1799. 18369141 10.1126/science.1152398

[73] Gasch AP, Moses AM, Chiang DY, Fraser HB, Berardini M, et al (2004) Conservation and evolution of *Cis*-regulatory systems in ascomycete fungi. PLoS Biol 2: e398. 15534694 10.1371/journal.pbio.0020398 PMC526180

[74] Park J, Park B, Jung K, Jang S, Yu K, et al (2008) CFGP: a web-based, comparative fungal genomics platform. Nucleic Acids Res 36: D562–D571. 17947331 10.1093/nar/gkm758 PMC2238957

[75] Bowden RL, Leslie JF (1999) Sexual recombination in *Gibberella zeae* . Phytopathology 89: 182–188. 18944794 10.1094/PHYTO.1999.89.2.182

