# Supplementary figures and images for: AbaA Regulates Conidiogenesis in the Ascomycete Fungus Fusarium graminearum
Source: PLoS One. 2013 Sep 10;8(9):e72915. doi: 10.1371/journal.pone.0072915 (PMC3769392; doi:10.1371/journal.pone.0072915)

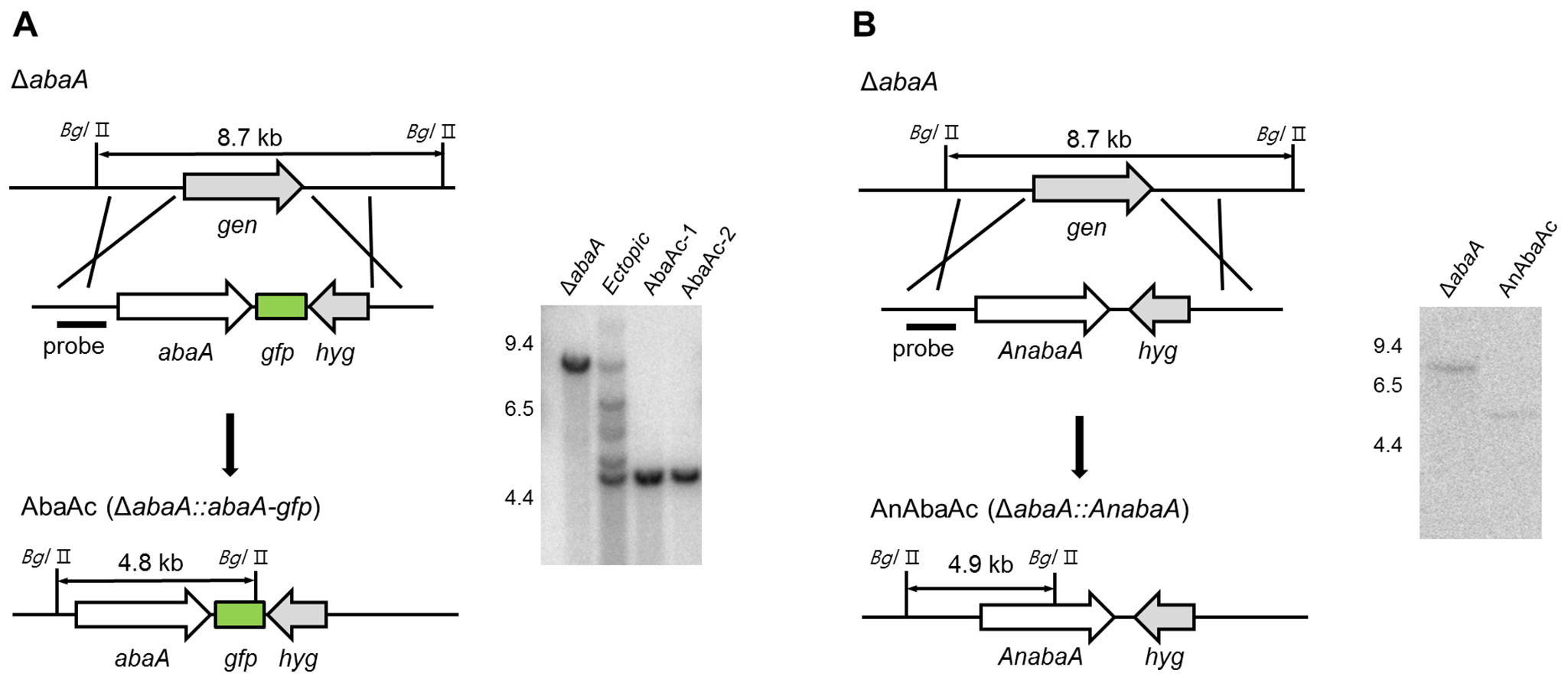

Supplement: Figure S1 — Complementation of abaA . Intra- (A) and interspecies (B) complementation of abaA in the abaA deletion mutant. ΔabaA, abaA deletion mutant; AbaAc, ΔabaA-derived strain complemented with abaA of F. graminearum; AnAbaAc, ΔabaA-derived strain complemented with abaA of A. nidulans (AnabaA); gen, geneticin resistance gene cassette; hyg, hygromycin B resistance gene cassette. The sizes of DNA standards (kb) are indicated on the left of the blot. (TIF) [file pone.0072915.s001.tif]

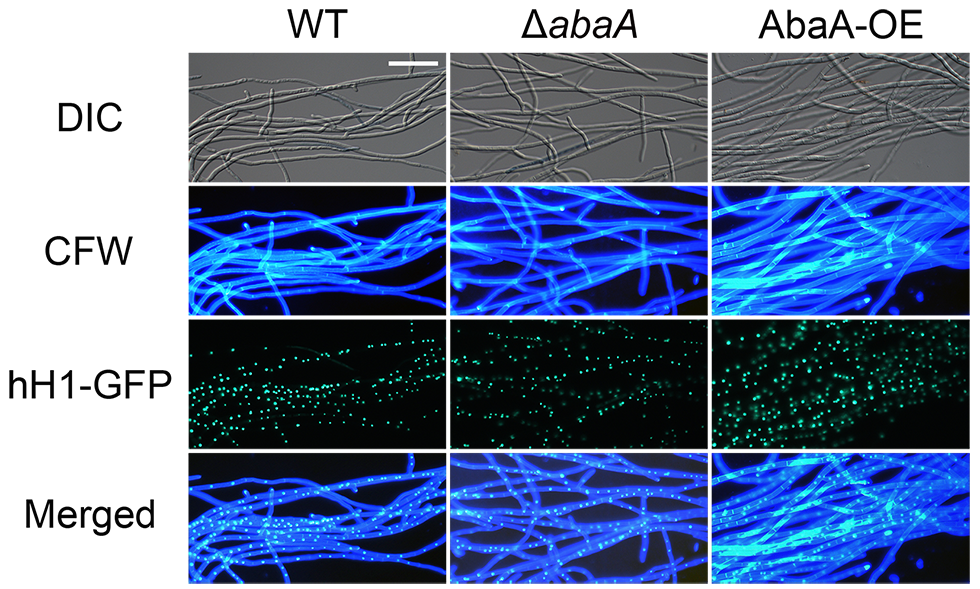

Supplement: Figure S2 — Mycelial morphology of F. graminearum strains. Differential interference contrast (DIC) and fluorescent microscopic observations were conducted 1 day after inoculation in complete medium (CM). Chitin accumulation in hyphae was visualized with Calcofluor white (CFW) staining. WT, hH1-GFP strain; ΔabaA, ΔabaA-g strain; AbaA-OE, AbaA-OE-g strain. Scale bar = 50 µm. (TIF) [file pone.0072915.s002.tif]
