# Supplementary material for: AbaA Regulates Conidiogenesis in the Ascomycete Fungus Fusarium graminearum
Source: PLoS One. 2013 Sep 10;8(9):e72915. doi: 10.1371/journal.pone.0072915 (PMC3769392; doi:10.1371/journal.pone.0072915)
Supplement: Table S1 — Primers used in this study. (PDF) [file pone.0072915.s003.pdf]

Table S1. Primers used in this study

Primer	Sequence (5'→3')	Description
AbaA-5F	AACCTCACTGTTTGTGCGGAACTTA	Forward and reverse primers for amplification of the 5'-flanking region of *abaA* with a tail for geneticin resistance gene cassette fusion
AbaA-5R	gcacaggtacacttgtttagagGTCAAGTCAGGTAAGGACGCTGTCAT	
AbaA-3F	ccttcaatatcatcttctgtcgTAACGCAGAGTGTGACACTGGCC	Forward and reverse primers for amplification of the 3'-flanking region of *abaA* with a tail for geneticin resistance gene cassette fusion
AbaA-3R	GTCGTCATCTCATGGTTTTTTATCGC	
AbaA-5N	CGAAGAAACCAGGGCAGACACTTA	Forward and reverse nest primers for third fusion PCR for amplification of the *abaA* deletion construct
AbaA-3N	TTGATCAAGCGCCCTAAATAACTCC	
Gen-for	CGACAGAAGATGATATTGAAGG	Forward and reverse primers for amplification of the geneticin cassette from the pII99 vector
Gen-rev	CTCTAAACAAGTGTACCTGTG	
pPRN3-N-For	GTCGAAAATTCAAGACAAGG	For RACE-PCR
pPRN3-N-Rev	AAGCGTGACATAACTAATTAC	For RACE-PCR
AbaA-RACE-1	AGTACCGCAACCGACAAGCACA	For RACE-PCR and sequencing of *abaA*
AbaA-RACE-2	TGCTGCTGTCATTGTTGGTGGTAT	
AbaA-RACE-3	GTCTCTTCTTCCGAGGTCAAGTTCAG	
AbaA-RACE-4	GATTGAAGGAGTAGGAGACATGATGGAG	
AbaA-RACE-5	ACACAATCACAATGGGCACAAATG	
AbaA-RACE-6	TTCTGGCGATTCAACACAACTAACC	
AbaA-RACE-7	CAACACAGATTGTGAAGATGTATTCGCTA	
AbaA-RACE-8	TTACACACTTCCAGCGGTGGCTT	
AbaA-RT-For	ACTCAGGAAGCTTTGACCACGGC	For real-time PCR of *abaA*
AbaA-RT-Rev	GGGCTCTGGTAGGGGTTGACAGTA	
WetA-RT-For	GTTCCAGGTACTCCCACTGCCAT	For real-time PCR of *wetA*
WetA-RT-Rev	ACGTTCTCGTCGCGCTTTGGT	
GzCyp1-RT-for	TCAAGCTCAAGCACACCAAGAAGG	For real-time PCR of *Gzcyp1*

GzCyp1-RT-rev	GGTCCGCCGCTCCAGTCT	
AbaA-5R OE	gatagtggaaaccgacgccccGAATTCAAGACGTGGAATGGTGGG	Reverse primer for amplification of the 5'-flanking region of *abaA* with a tail for *ef1α* promoter replacement
AbaA-3F OE	tatcacaaaaggaacccaatcttcaaagACAAACCGTCGCCATGTCTTCA	Forward and reverse primers for amplification of the 3'-flanking region of *abaA* with a tail for *ef1α* promoter replacement
AbaA-3R OE	CCTGAGTACGACCCTTGTAGACC	
AbaA-3N OE	GTAAGTCCATTGTTTGTTGCAGTG	Reverse nest primer for amplification of the *AbaA* promoter replacement with the *ef1α* promoter
Neo-For new	GGGGCGTCGGTTTCCACTATC	Forward and reverse primers for amplification of *gen*-$P_{ef1\alpha}$ from the pSKGEN vector
EF Pro-Rev new	CTTTGAAGATTGGGTTCCTTTTGTGATA	
Neo-For 5N	GGCGAGTACTGCCAGCAGTAGACAC	Forward nest primer for amplification of the *abaA* promoter replacement with the *ef1α* promoter
AbaA-5R AnabaA	aattccaaagacgagatattgagGGGAGCTGGAAGGCTGGC	Primers used for interspecies complementation using *A. nidulans* AbaA
AnAbaA-For	CTCAATATCTCGTCTTTGGAATT	
AnabaA-Rev hyg	tgagacaaatggtgttcaggatctcTTATGAATTAAGAGGTTCTCCCCTG	
pBCATPH-comp-3 R	GAGATCCTGAACACCATTTGTCTCA	
